# Molecular Detection of *Balantioides coli* in Pigs Employing the Traditional PCR and a Novel Cross‐Priming Amplification‐Based Lateral Flow Assay

**DOI:** 10.1155/tbed/4982345

**Published:** 2026-07-10

**Authors:** Kui-Hao Liu, Ze-Dong Zhang, Yu-Xuan Wang, Zi-Rui Wang, Wen-Wei Gao, Xing-Quan Zhu, Qing Liu

**Affiliations:** ^1^ College of Veterinary Medicine, Shanxi Agricultural University, Taigu, 030801, Shanxi Province, China, sxau.edu.cn

**Keywords:** *Balantioides coli*, cross-priming amplification, detection method, genetic diversity, molecular prevalence

## Abstract

*Balantioides coli* is the only ciliate with significant implications for both human and animal health. Regarding animals, *B. coli* primarily infects pigs. However, there is a lack of data regarding *B. coli* in pigs in Shanxi, a province in northern China covering 156,700 km^2^ where pig farming is a major agricultural industry. In this study, 341 pig fecal samples collected from this province were examined for the presence of *B. coli* using a traditional polymerase chain reaction (PCR) method targeting the β‐tubulin gene. The results showed that the overall prevalence of *B. coli* in the sampled pigs was 75.1%, and its prevalence varied significantly across different regions. Among the polymorphic sequences identified in this study, the genotype with zoonotic potential (genotype I) was observed. In addition, we established a cross‐priming amplification (CPA) strategy–assisted lateral flow immunoassay (LFIA) biosensor for the detection of *B. coli*. The whole process from amplification to visual readout can be completed within 63 min without any special instrument. The biosensor exhibited a sensitivity of 100 copies/µL of plasmid DNA and specifically recognized *B. coli*. The results of the diagnostic performance analysis revealed that the biosensor accurately distinguished between PCR‐positive and ‐negative fecal samples. Our results contribute to expanding our understanding of the geographical distribution and genetic diversity of *B. coli* in pigs and have important implications for controlling *B. coli* infection in pigs and humans. Also, this study provides a novel molecular tool for the detection of *B. coli*, particularly in resource‐limited settings.

## 1. Introduction

*Balantioides coli* is a ciliated protist with a broad host range, including pigs, nonhuman primates, cattle, donkeys, birds, rodents, sheep, goats, and humans [1–3]. It mainly inhabits its host’s intestinal tract and has a direct life cycle, and the fecal‐oral route serves as the mode of transmission [1]. The life cycle of *B. coli* consists of two stages (the active trophozoite colonizing the intestine and the dormant cyst), and cysts are considered the main stage of infection [1]. Infection in humans and animals can occur through direct contact with infected subjects or indirect ingestion of cyst‐contaminated food or water [1, 4].

In humans, infection can be asymptomatic and symptomatic. Clinical manifestations, when present, may be chronic balantidiasis characterized by colitis with unspecific abdominal disorders or acute symptoms (a dysenteric form) [1]. Severe or fatal infection may occur in debilitated/immunocompromised hosts. In addition, the parasite can disseminate to extraintestinal sites, including the urogenital tract and lungs [5–7]. In pigs, the involvement of *B. coli* in diarrhea and enteritis has been described [8].

Notably, human infection with *B. coli* is more frequent where close contact occurs between humans and animals, particularly pigs [9]. Hence, understanding the epidemiology of *B. coli* in pigs is of great importance for two main reasons: (i) it provides a basis for the implementation of effective prevention and control strategies in pigs, thereby reducing economic losses; and (ii) it helps prevent human infection with *B. coli* resulting from contact with pigs. Unfortunately, though pig farming is a key part of animal husbandry in Shanxi Province, northern China, no epidemiological data on *B. coli* in pigs are yet available for this province.

Because their sizes and morphological characteristics are distinctive, *B. coli* trophozoites and cysts can be identified in feces by optical microscopy, which is used for epidemiological detection [4, 10]. However, compared with the traditional method, polymerase chain reaction (PCR)–based assays have advantages, such as enabling the concurrent analysis of both the prevalence and genetic characteristics of *B. coli* [11]. For example, through PCR amplification and sequencing of the ITS1‐5.8S rRNA‐ITS2 (ITS) region, two main genetic types (A and B) were differentiated [12]. More recently, based on the β‐tubulin gene, a novel nested PCR assay was developed for the detection and molecular characterization of *B. coli* [13].

Of course, these detection methods involve complex procedures or require expensive equipment, thus limiting their practicality for field use. Compared to the traditional PCR methods, isothermal amplification methods offer the advantage of speed, eliminating the need for a thermal cycler [14–17]. Cross‐priming amplification (CPA), an isothermal nucleic acid amplification method, is increasingly utilized in diagnostics due to its high sensitivity and performance [18–20]. For instance, it can detect as few as four bacterial cells [21]. Therefore, the development of a method based on the CPA technique for the detection of *B. coli* warrants further investigation.

In the present study, the prevalence of *B. coli* in pigs in different factors (region, age, and sex) in Shanxi Province was investigated by using the PCR‐based method. Meanwhile, the genetic diversity of *B. coli* obtained in the present study was investigated by analyzing the β‐tubulin gene. Furthermore, an assay based on CPA was developed to detect *B. coli*. To facilitate rapid visual detection, the amplification products were analyzed using lateral flow immunoassay (LFIA) strips. The efficacy of the method was verified by testing its specificity and sensitivity, and its practicality was further assessed using a number of pig fecal samples.

## 2. Materials and Methods

### 2.1. Sampling and DNA Extraction

The sampling was carried out in November 2020. In total, 341 fresh fecal samples were collected from pig farms in the northern (Shanyin County, small‐scale farms, *n* = 110), central (Qi County, small‐scale farms, *n* = 48), and southern (Jishan County, large‐scale farms, *n* = 183) parts of Shanxi Province. At the sampling time, age information for each animal was recorded by interviewing the farmer (≤4 months, *n* = 96; >4 and to 6 months, *n* = 131; and >6 months, *n* = 114). Each fecal sample was promptly collected from the upper portion of fresh droppings by using disposable gloves. All fecal samples were kept in cool boxes with ice packs, promptly transported to our laboratory, and subsequently maintained in a refrigerator at −20°C until further use. Following the manufacturer’s protocol, total genomic DNA of each frozen fecal sample was extracted with the EZNA Stool DNA extraction kit (Omega Bio‐tek Inc., Norcross, GA, USA). DNA extracts were frozen at −20°C for further molecular analysis.

### 2.2. PCR Amplification and Sequencing

To determine the prevalence of *B. coli*, all DNA samples were screened by nested PCR amplification of the β‐tubulin gene using published primers (F1: 5′‐AACTGGGCTAAGGGACACTA‐3′, R1: 5′‐CTCCATTTCGTCCATACCTT‐3′; F2: 5′‐GACCTTCGCTGTCTTCCC‐3′, R2: 5′‐TTCTCCGGTGTACCAATGT‐3′) and a thermal profile [13]. PCR products were resolved on ethidium bromide‐stained 1.5% (wt/vol) agarose gels, and amplicons were visualized under UV illumination. Then, the desired amplicon size was sliced, purified, and sent to Sangon Biotech Co., Ltd. (Shanghai, China) for sequencing in both directions using the same primers for PCR amplification with a 3730XL DNA analyzer (Applied Biosystems, Thermo Fisher Scientific, Waltham, MA, USA).

### 2.3. Sequencing and Phylogenetic Analyses

Sequence editing and assembly were carried out using Chromas Pro v2.1.3 (Technelysium Pty Ltd., Tewantin, Queensland, Australia). After processing, the final nucleotide sequences were compared with those available in the GenBank database using the Basic Local Alignment Search Tool (BLAST) (http://www.ncbi.nlm.nih.gov/blast/) to identify known closely related sequences. To analyze the sequence variations, the sequences generated in the present study were analyzed with the BioEdit Sequence Alignment Editor version 7.7.1.0 and compared with reference sequences (PV609776–PV609780) deposited in the NCBI GenBank database. Phylogenetic trees were constructed by the neighbor‐joining (NJ) method using the MEGA 7 software package (http://www.megasoftware.net/), and distance analysis was performed according to the Kimura 2–parameter model [22, 23]. Tree topology reliability was assessed by bootstrapping with 1000 replicates. iTOL was used to visualize phylogenetic trees.

### 2.4. Construction of the Recombinant Plasmid

An 1640‐bp fragment of the 18S rRNA gene of *B. coli* was amplified using PCR with specific primers (forward: 5′‐AACCTGGTTGATCCTGCCAGT‐3′ and reverse: 5′‐TGATCCTTCYGCAGGTTCACCTACGG‐3′) [24]. PCR products were cloned into the pMD18‐T vector (Takara, Dalian, China) to construct the final plasmid (designated PMD19‐18S). A NanoDrop spectrophotometer (Thermo Fisher Scientific, Waltham, USA) was used to measure the concentration of plasmid PMD18‐18S, and its copy number was calculated according to the following formula:

6.02×1023×plasmid concentration in grams per microliter/plasmid length in base pairs×660.

### 2.5. Optimization of the CPA Primers and Reaction Temperature

To obtain a primer set of high performance, four distinct sets of five primers were designed with the online software PrimerExplorer V5 (http://primerexplorer.jp/e/) targeting the 18S rRNA gene of *B. coli*, followed by primer synthesis from Tsingke Biotechnology Co., Ltd. (Beijing, China). Table 1 provides the PCR primer sequences. Regarding primer set screening, each CPA reaction was prepared as follows: 8 U of Bst DNA polymerase (New England Biolabs, Ipswich, MA, USA), 1 mM dNTPs (New England Biolabs, Ipswich, MA, USA), 0.4 µM each of two CPA primers (Primer 1 and Primer 2), 0.6 µM each of three CPA primers (Primer 3, Primer 4, and Primer 5), 0.5 M of betaine (Merck KGaA, Darmstadt, Germany), 4 mM MgSO_4_, 1× Bst buffer, 1 μL of template DNA or nuclease‐free water (Solarbio, Beijing, China), and an appropriate volume of nuclease‐free water (Solarbio, Beijing, China) to reach a final reaction volume of 25 µL. The optimal primer set was identified through CPA reactions carried out at 59°C for 1 h. Following the selection, temperature optimization was carried out by incubating the reaction mixtures for 1 h at constant temperatures from 55 to 63°C in 2°C intervals.

**Table 1:** Primer information.

Primer sets	Primers	Sequences (5′ → 3′)
BC1	BC1‐1	biotin‐AATTGTCAGAGGTGAAATTCTTGGA
	BC1‐2	FITC‐GTTAAAGACTAACGTATGCGAAAGC
	BC1‐3	GCATGGAATAACGAATGTGT
	BC1‐4	AATTGTCAGAGGTGAAATTCTTGGATGAAAACATCCTTGGCAAAT
	BC1‐5	TCCCCTATCTTTCGTTCTTG
BC2	BC2‐1	biotin‐GTGTTTCAAGCAGGCTTTTGCA
	BC2‐2	FITC‐AGCATGGAATAACGAATGTGTCTAG
	BC2‐3	ATTTCAAGGCGTGTATACTCT
	BC2‐4	GTGTTTCAAGCAGGCTTTTGCATCGCAATCTAGAATTAACCAAG
	BC2‐5	GTTGGGGGCATTAGTATTTAA
BC3	BC3‐1	biotin‐TGGTCGCAAGACTGAAACTTAAAG
	BC3‐2	FITC‐GCACCACCAGGAGTGGA
	BC3‐3	GTAGTCCTATCTATAAACTATGCCG
	BC3‐4	TGGTCGCAAGACTGAAACTTAAAGTGTTGAGTCAAATTAAGCCG
	BC3‐5	ACCTGGTAAGTTTCCCCG

The amplification products were analyzed either by agarose gel electrophoresis or a LFIA. For LFIA, the amplification products were thoroughly mixed with 70 μL of HybriDetect Assay Buffer (Milenia Biotec GmbH, Giessen, Germany). Then, the test strip (Milenia Biotec GmbH, Giessen, Germany) was inserted into the mixture containing the DNA product and buffer, and the results were read after 3‐min incubation at room temperature. If the first detection line displays a band, the result is positive. The image of each test strip was immediately collected after 3‐min incubation using a smartphone and further processed with ImageJ software by converting it to 8‐bit grayscale to extract the gray values of both the first and second lines for the calculation of their ratio [25].

### 2.6. Sensitivity and Cross‐Reaction of the CPA‐LFIA Biosensor

To assess the sensitivity, 10‐fold serially diluted pMD18‐18S plasmid DNA (1000 to 1 copies/μL) was analyzed by PCR and the CPA‐LFIA biosensor. Then, a comparative analysis was performed. To assess the cross‐reactivity of the CPA‐LFIA biosensor, different genomic DNA templates of other parasites from previous studies conducted in our laboratory were used for analysis; the parasites included *Blastocystis* sp., *Giardia duodenalis*, *Enterocytozoon bieneusi*, *Cryptosporidium* sp., *Tritrichomonas foetus*, *Tetratrichomonas buttreyi*, and *Entamoeba* sp. [26–31].

### 2.7. Applicability of the CPA‐LFIA Biosensor

To evaluate the diagnostic performance of the CPA‐LFIA biosensor, a validation study was conducted using 36 pig fecal samples. These samples were among those used in the prevalence study mentioned above and have been examined using the β‐tubulin–based nested PCR method. All samples were tested with the CPA‐LFIA biosensor. The final step involved a comparative analysis to determine the concordance between the visual readouts from the lateral flow strips and the PCR results, thereby assessing the accuracy and reliability of the CPA‐LFIA biosensor.

### 2.8. Statistical Analysis

In the present study, descriptive analysis of data obtained from the survey was performed with SPSS 26.0 software (IBM, Chicago, IL, USA). Briefly, a chi‐square analysis (in the menu bar, click Analyze → Descriptive Statistics → Crosstabs… → Statistics → Chi‐square) was carried out to examine the relationship between prevalence and each of the three factors (region, age, and sex). Odds ratios (ORs) and corresponding 95% confidence intervals (CIs) (in the menu bar, click Analyze → Descriptive Statistics → Crosstabs… → Statistics → Risk) were calculated to assess the strength of the associations observed. The threshold for statistical significance was set at a *p*‐value of 0.05.

Data for gray values obtained from lateral flow assays were expressed as mean ± standard deviation (SD), and mean values were analyzed using a one‐way analysis of variance (in the menu bar, click Analyze → Compare Means → One‐Way ANOVA…) with SPSS 26.0 software (IBM, Chicago, IL, USA). Post hoc comparisons were completed using Tukey–Kramer multiple comparison tests. The threshold for statistical significance was set at a *p*‐value of 0.05. GraphPad Prism version 8.0 (GraphPad Software, San Diego, CA, USA) was used for visualization.

## 3. Results

### 3.1. Prevalence of *B. coli* in Pigs Revealed by the Traditional PCR Targeting the β‐Tubulin Gene

Among the 341 pig fecal samples analyzed, 256 (75.1%, 95% CI: 70.1–79.6) were PCR‐positive for *B. coli* (Table 2). A significant difference in the prevalence of *B. coli* was observed among the three counties (*p* < 0.001). Qi County exhibited the highest infection rate at 85.4% (41/48), followed by Shanyin County at 83.6% (92/110) and Jishan County at 67.2% (123/183). Differences in prevalence among age groups were observed, with the highest in pigs ≤ 4 months (79.2%, 95% CI: 69.8–86.7) and the lowest in pigs aged over 4 and up to 6 months (70.2%, 95% CI: 61.8–77.7). Despite these differences, the variations did not reach statistical significance (*p* > 0.05). The prevalence of *B. coli* in female pigs (77.6%, 97/125) was higher than that in males (73.6%, 159/216), but the difference was not statistically significant.

**Table 2:** Prevalence of *Balantioides coli* in pigs by region, age, and sex.

Factor	Category	Number tested	Number positive	Prevalence % (95% CI)	OR (95% CI)	*p*‐Value
Region	Jishan	183	123	67.2 (60.2–73.7)	Reference	<0.001
	Qi	48	41	85.4 (72.2–93.9)	2.9 (1.2–6.8)	—
	Shanyin	110	92	83.6 (75.5–89.9)	2.5 (1.4–4.5)	—
Age	Month ≤ 4	96	76	79.2 (69.8–86.7)	1.6 (0.9–3.0)	—
	4 < Month ≤ 6	131	92	70.2 (61.8–77.7)	Reference	0.251
	Month > 6	114	88	77.2 (68.5–84.4)	1.4 (0.8–2.6)	—
Sex	Male	216	159	73.6 (67.2–79.4)	Reference	0.412
	Female	125	97	77.6 (69.4–84.5)	1.2 (0.7–2.1)	—
Total	—	341	256	75.1 (70.1–79.6)	—	—

### 3.2. Sequence Alignment

**Figure 1:**
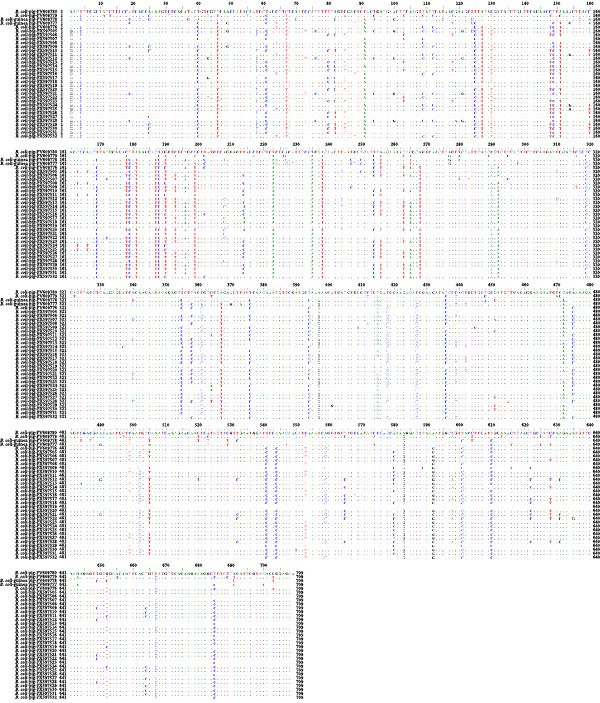
The partial β‐tubulin gene sequences (PX597505–PX597532) obtained in the present study were aligned with each other and the reference sequences (PV609776–PV609780). Dots indicate identical bases.

Sequencing analysis of the β‐tubulin gene yielded 28 distinct sequences, and the levels of sequence identity ranged from 89.8% to 99.9% (Figure 1). All the sequences obtained in the present study have been submitted to the NCBI GenBank database (accession numbers: PX597505–PX597532) and are publicly available. Among the three counties, 5 (PX597512–PX597514, PX597522, and PX597528), 7 (PX597505–PX597511), and 16 (PX597515–PX597521, PX597523–PX597527, and PX597529–PX597532) sequence variants were detected in Shanyin, Jishan, and Qi, respectively (Supporting Information 1: Table S1). Regarding age groups, 7 (PX597519, PX597521, PX597524, PX597526–PX597528, and PX597531), 12 (PX597505, PX597508–PX597511, PX597513, PX597514, PX597516, PX597520, PX597522, PX597523, and PX597530), and 9 (PX597506, PX597507, PX597512, PX597515, PX597517, PX597518, PX597525, PX597529, and PX597532) sequence variants were detected in pigs aged ≤4 months, >4 and to 6 months, and > 6 months, respectively (Supporting Information 1: Table S1). In addition, 16 (PX597505, PX597508–PX597511, PX597513, PX597514, PX597519–PX597521, PX597523, PX597524, PX597526–PX597528, and PX597530) and 12 (PX597506, PX597507, PX597512, PX597515, PX597516–PX597518, PX597522, PX597525, PX597529, PX597531, and PX597532) sequence variants were detected in males and females, respectively (Supporting Information 1: Table S1). One sequence (PX597519) shared 99.4% homology with the pig‐derived sequence (PV609780) from China. The remaining 27 sequences displayed high similarity to the pig‐derived sequence (PV609776) from China.

The 28 distinct sequences were translated into amino acid sequences. Amino acid sequence alignment showed that the majority of polymorphic sites corresponded to synonymous mutations, and only four positions exhibited nonsynonymous substitutions (Supporting Information 2: Figure S1). Specifically, amino acid differences were identified at positions 40 (glutamic acid, E vs. lysine, K), 135 (methionine, M vs. valine, V), 152 (phenylalanine, F vs. isoleucine, I), and 212 (M vs. V).

### 3.3. Phylogenetic Relationships

**Figure 2:**
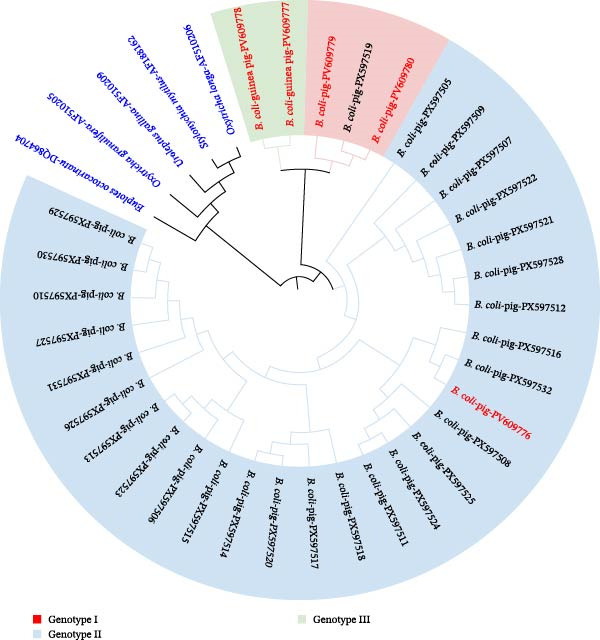
Phylogenetic relationships inferred by a neighbor‐joining analysis of the β‐tubulin gene sequences. The β‐tubulin gene sequences of *B. coli* obtained in the present study were displayed in black.

Based on the phylogenetic analysis using the β‐tubulin gene sequence data, *B. coli* isolated from pigs showed considerable genetic diversity (Figure 2). Though the 28 sequences generated in the present study did not exhibit 100% identity with the five available β‐tubulin gene sequences of *B. coli* in the NCBI GenBank database, the phylogenetic analysis unequivocally confirmed their classification as *B. coli* (Figure 2). Notably, one sequence (PX597519) obtained from one fecal sample was classified into genotype I, and the remaining 27 different sequences detected in 255 fecal samples were classified into genotype II. The simultaneous presence of genotypes I and II in a single sample was not observed.

### 3.4. Primer Screening for the CPA‐LFIA Biosensor

**Figure 3:**
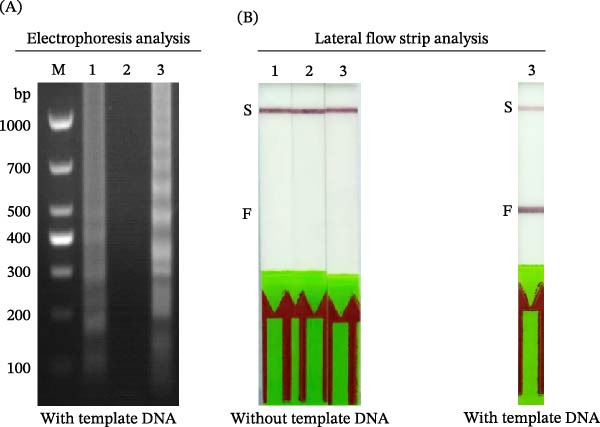
Screening of the optimal primer set using agarose gel electrophoresis and lateral flow strips. (A) PCR products amplified by the corresponding primer sets were subjected to agarose gel electrophoresis. Lane M denotes DNA marker. Lanes 1–3: the primer sets BC1–3. (B) CPA products amplified with the corresponding primer sets, in the presence or absence of template DNA, were analyzed using lateral flow strips. F: first detection line; S: second detection line. Lanes 1–3: the primer sets BC1–3.

For the development of the CPA‐LFIA biosensor, three CPA primer sets (BC1, BC2, and BC3) were designed and tested using both agarose gel electrophoresis and lateral flow strips. Assessment by agarose gel electrophoresis demonstrated that BC1 and BC3 generated many bands of different sizes, whereas BC2 yielded no amplicons (Figure 3A). Negative controls (without template DNA) were included, and no positive results were observed (Figure 3B). Meanwhile, LFIA confirmed successful amplification with BC3 (Figure 3B). Consequently, BC3 was chosen for further analysis due to its optimal performance (see Table 1 for primer details).

### 3.5. Screening of Optimal Reaction Temperature for the CPA‐LFIA Biosensor

**Figure 4:**
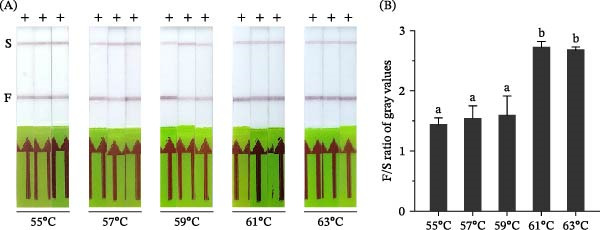
(A) CPA reactions were performed at 55, 57, 59, 61, and 63°C to screen the optimal temperature. F: first detection line; S: second detection line. “+” indicates a positive result. (B) The ratio of gray values between F line and S line of lateral flow strips in (A). The data shown represent the mean ± standard deviation (SD) from three independent experiments. Bars sharing different letters are significantly different.

To optimize the reaction temperature for the CPA‐LFIA biosensor, assays were conducted at a range of 55–63°C using the optimal primer set (BC3) (all CPA reactions described in the subsequent sections were performed using this primer set). Following a 60‐min incubation, results analyzed using lateral flow strips indicated that 61°C enabled the most robust amplification (Figure 4).

### 3.6. Evaluation of the Sensitivity and Specificity of the CPA‐LFIA Biosensor

**Figure 5:**
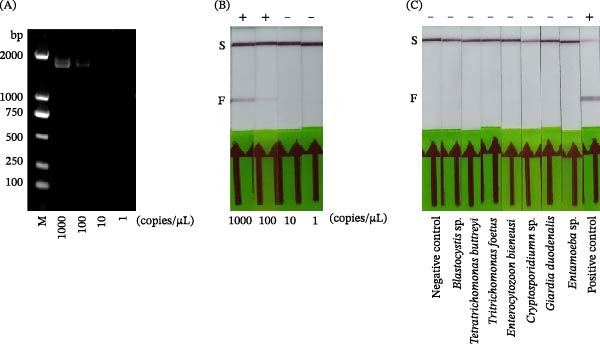
Analysis of the sensitivity and specificity of the CPA‐LFIA biosensor. (A) A 10‐fold serial dilution of the recombinant plasmid pMD18‐18S was analyzed by PCR. (B) A 10‐fold serial dilution of the recombinant plasmid pMD18‐18S was analyzed by the biosensor. (C) Specificity evaluation of the biosensor using DNA samples of *Blastocystis* sp., *Giardia duodenalis*, *Enterocytozoon bieneusi*, *Cryptosporidium* sp., *Tritrichomonas foetus*, *Tetratrichomonas buttreyi*, and *Entamoeba* sp. F: first detection line; S: second detection line. “+” indicates a positive result, and “−” indicates a negative result. Negative control: without target DNA. Positive control: with target DNA.

Sensitivity testing using 10‐fold serially diluted pMD18‐18S plasmid DNA showed that the detection limit of the PCR method was 100 copies/µL of plasmid DNA (Figure 5A). Also, the CPA‐LFIA biosensor achieved the same detection sensitivity (Figure 5B). Specificity testing demonstrated that the CPA‐LFIA biosensor accurately discriminated its target (*B. coli*) from nontarget species (*Blastocystis* sp., *G. duodenalis*, *E. bieneusi*, *Cryptosporidium* sp., *T. foetus*, *T. buttreyi*, and *Entamoeba* sp.) (Figure 5C).

### 3.7. Evaluation of the Biosensor in Practical Applications

**Figure 6:**
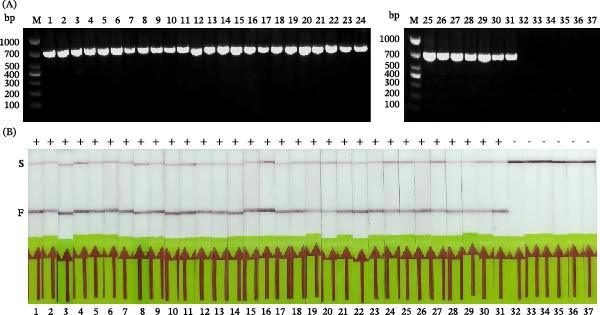
Practical application of the biosensor for sample detection. (A) A total of 36 pig fecal samples were analyzed by PCR. Number 37 represents the negative control without target DNA. (B) The 36 pig fecal samples were also analyzed by the biosensor. Number 37 represents the negative control without target DNA. F: first detection line; S: second detection line. “+” indicates a positive result, and “−” indicates a negative result.

To evaluate the performance of the CPA‐LFIA biosensor, a total of 36 pig fecal samples were included for analysis. PCR analysis showed that 31 tested positive, and five were negative (Figure 6A). The biosensor correctly identified all positive and negative samples, showing 100% concordance with the conventional PCR (Figure 6B).

## 4. Discussion

*B. coli* is a globally distributed parasite, primarily infecting pigs [3]. Previous studies have shown highly variable prevalence rates in pigs, ranging from 0% to 100% [32–35]. Differences in prevalence may be affected by several factors, such as diagnostic methods. Compared with microscopic examinations using the fecal flotation technique with sodium nitrate, a previous study reported that molecular methods generally show higher sensitivity [36]. Though another study showed that microscopic examinations with the sedimentation technique showed high sensitivity [11], the PCR‐based method enables the concurrent analysis of both the prevalence and genetic characteristics of *B. coli*. Hence, in the present study, we conducted a prevalence assessment of *B. coli* in pigs in Shanxi Province using the traditional PCR to target the β‐tubulin gene region, which was reported to be a robust marker for epidemiological studies of *B. coli* [13]. The overall prevalence of *B. coli* in pigs in Shanxi Province, 75.1%, was rather high. Considerable geographical heterogeneity in the prevalence of *B. coli* was found in the present study, which has also been reported in previous studies carried out using traditional PCR assays [35, 36].

In addition to being able to identify *B. coli*, PCR‐based methods combined with sequencing and phylogenetic analysis have allowed for the investigation of its different molecular characteristics. Through phylogenetic analysis of the β‐tubulin gene sequences, *B. coli* were categorized into three distinct genotypes (I–III) [13]. Genotypes I and II exhibit potential for cross‐species transmission, while genotype III displays strict host specificity [13]. Through phylogenetic analysis of the ITS1‐5.8S‐ITS2 region, *B. coli* were categorized into two main types of sequence variants, namely, A and B [12]. In the present study, two genotypes (I and II) were identified through phylogenetic analysis of the β‐tubulin gene sequences. ITS types A and B match β‐tubulin genotypes I and II, respectively [13], and type A has zoonotic potential [12]. This indicated that pigs in the investigated regions may transmit *B. coli* to diverse hosts (including humans), thereby causing a burden to human and animal health. Sequencing analysis identified novel sequences in genotypes I and II, expanding the knowledge on the genetic divergence of *B. coli*. Nevertheless, high‐frequency synonymous mutations were observed, which were consistent with the results of the previous study [13].

Isothermal amplification techniques are emerging as promising alternatives to traditional PCR methods for detecting various pathogens [37]. In the present study, CPA was chosen for several reasons. For example, it requires only an enzyme with strand displacement activity and a set of five primers [38]. Primers and reaction temperatures are pivotal parameters that significantly impact the efficiency of nucleic acid amplification [37]. The presence of multiple copies of the 18S rRNA gene in eukaryotic genomes makes it a highly sensitive marker for detection assays [39]. Based on the 18S rRNA gene of *B. coli*, one satisfactory primer set (BC3) used for CPA was obtained in the present study. Subsequently, this primer set demonstrated robust amplification efficiency at 61°C.

A previously reported PCR‐based method targeting the ITS1‐5.8S rRNA‐ITS2 gene region for the detection of *B. coli* required ~130 min, which included about 105 min for PCR amplification and an additional ~25 min for agarose gel electrophoresis [40]. The PCR‐based method targeting the β‐tubulin gene for the detection of *B. coli* required ~227 min, which included about 202 min for PCR amplification and an additional ~25 min for agarose gel electrophoresis [13]. The novel CPA assay established in the present study enabled the rapid detection of *B. coli* in 60 min. Hence, in addition to the advantage of no need for an expensive thermal cycler, the method established in the present study provides faster results compared with the traditional PCR‐based methods. However, the CPA method does not provide sequence information, making it unsuitable for genetic diversity analysis of *B. coli*.

In addition to *B. coli*, several parasites inhabit the digestive tracts of pigs, from where they are expelled into the environment with feces [30, 41–44]. Hence, several intestinal protozoan species were included to assess the specificity of the CPA assay, including *Blastocystis* sp., *G. duodenalis*, *E. bieneusi*, *Cryptosporidium* sp., *T. foetus*, *T. buttreyi*, and *Entamoeba* sp. The CPA assay enabled the distinct identification of *B. coli*, as evidenced by the absence of cross‐reactivity with other related protozoan DNA samples. Furthermore, the CPA‐LFIA biosensor achieved the same detection sensitivity as the PCR method with 100 copies/μL of plasmid DNA.

Diagnostic performance analysis revealed complete concordance between the CPA assay and the PCR‐based method for all positive and negative samples, indicating its potential as a new molecular tool for the identification of *B. coli*, particularly in areas with limited laboratory infrastructure or in pig farms. Nevertheless, the roles of the traditional PCR‐based methods remain irreplaceable, which continue to be vital for the genotyping of *B. coli*.

## 5. Conclusion

Using the traditional PCR‐based method targeting the β‐tubulin gene region, the present study revealed the *B. coli* prevalence of 75.1% in pigs in Shanxi Province; subsequently, its prevalence was found to be significantly different among different regions. PCR amplification combined with sequence analysis of the β‐tubulin gene identified the genotype with zoonotic potential. This indicated the possibility of transmission between pigs and humans, though the genotype with zoonotic potential was only detected in one sample. In addition, we developed a novel CPA‐assisted biosensor for the rapid detection of *B. coli*, showing several advantages over the traditional PCR‐based methods. The traditional PCR‐based methods together with the novel CPA‐assisted biosensor are expected to enhance the detection and control of *B. coli* infection in humans and pigs.

## Funding

This work was supported by the Research Fund of Shanxi Province for Introduced High‐level Leading Talents (Grant RFSXIHLT202101) and the Special Research Fund of Shanxi Agricultural University for High‐level Talents (Grant 2021XG001).

## Disclosure

All authors have agreed to be accountable for all aspects of the work and approved the final version of the manuscript.

## Ethics Statement

The samples examined in this study were fecal samples, the collection of which did not require an ethical approval.

## Conflicts of Interest

The authors declare no conflicts of interest.

## Supporting Information

Additional supporting information can be found online in the Supporting Information section.

## Supporting information


**Supporting Information 1** Table S1: Sequence distribution by region, sex, and age.

**Supporting Information 2** Figure S1: Alignment of β‐tubulin amino acid sequences.

## Data Availability

The data that support the findings of this study are openly available in GenBank at https://www.ncbi.nlm.nih.gov/genbank/, Reference Number PX597505–PX597532.
