# Supplementary material for: Molecular Detection of Balantioides coli in Pigs Employing the Traditional PCR and a Novel Cross‐Priming Amplification‐Based Lateral Flow Assay
Source: Transbound Emerg Dis. 2026 Jul 10;2026:4982345. doi: 10.1155/tbed/4982345 (PMC13351935; doi:10.1155/tbed/4982345)
Supplement: Supplementary file 1 — Supporting Information 1 Table S1: Sequence distribution by region, sex, and age. [file TBED-2026-4982345-s001.docx]

| **Sequence** | **Region** | **Sex** | **Age** |
| --- | --- | --- | --- |
| PX597505 | JiShan | Male | 4 < Month ≤ 6 |
| PX597506 | JiShan | Female | Month > 6 |
| PX597507 | JiShan | Female | Month > 6 |
| PX597508 | JiShan | Male | 4 < Month ≤ 6 |
| PX597509 | JiShan | Male | 4 < Month ≤ 6 |
| PX597510 | JiShan | Male | 4 < Month ≤ 6 |
| PX597511 | JiShan | Male | 4 < Month ≤ 6 |
| PX597512 | ShanYin | Female | Month > 6 |
| PX597513 | ShanYin | Male | 4 < Month ≤ 6 |
| PX597514 | ShanYin | Male | 4 < Month ≤ 6 |
| PX597515 | Qi | Female | Month > 6 |
| PX597516 | Qi | Female | 4 < Month ≤ 6 |
| PX597517 | Qi | Female | Month > 6 |
| PX597518 | Qi | Female | Month > 6 |
| PX597519 | Qi | Male | Month ≤ 4 |
| PX597520 | Qi | Male | 4 < Month ≤ 6 |
| PX597521 | Qi | Male | Month ≤ 4 |
| PX597522 | ShanYin | Female | 4 < Month ≤ 6 |
| PX597523 | Qi | Male | 4 < Month ≤ 6 |
| PX597524 | Qi | Male | Month ≤ 4 |
| PX597525 | Qi | Female | Month > 6 |
| PX597526 | Qi | Male | Month ≤ 4 |
| PX597527 | Qi | Male | Month ≤ 4 |
| PX597528 | ShanYin | Male | Month ≤ 4 |
| PX597529 | Qi | Female | Month > 6 |
| PX597530 | Qi | Male | 4 < Month ≤ 6 |
| PX597531 | Qi | Female | Month ≤ 4 |
| PX597532 | Qi | Female | Month > 6 |

**Table S1** Sequence distribution by region, sex, and age.
