# Supplementary material for: Molecular Detection of Balantioides coli in Pigs Employing the Traditional PCR and a Novel Cross‐Priming Amplification‐Based Lateral Flow Assay
Source: Transbound Emerg Dis. 2026 Jul 10;2026:4982345. doi: 10.1155/tbed/4982345 (PMC13351935; doi:10.1155/tbed/4982345)
Supplement: Supplementary file 2 — Supporting Information 2 Figure S1: Alignment of β‐tubulin amino acid sequences. [file TBED-2026-4982345-s002.pdf]

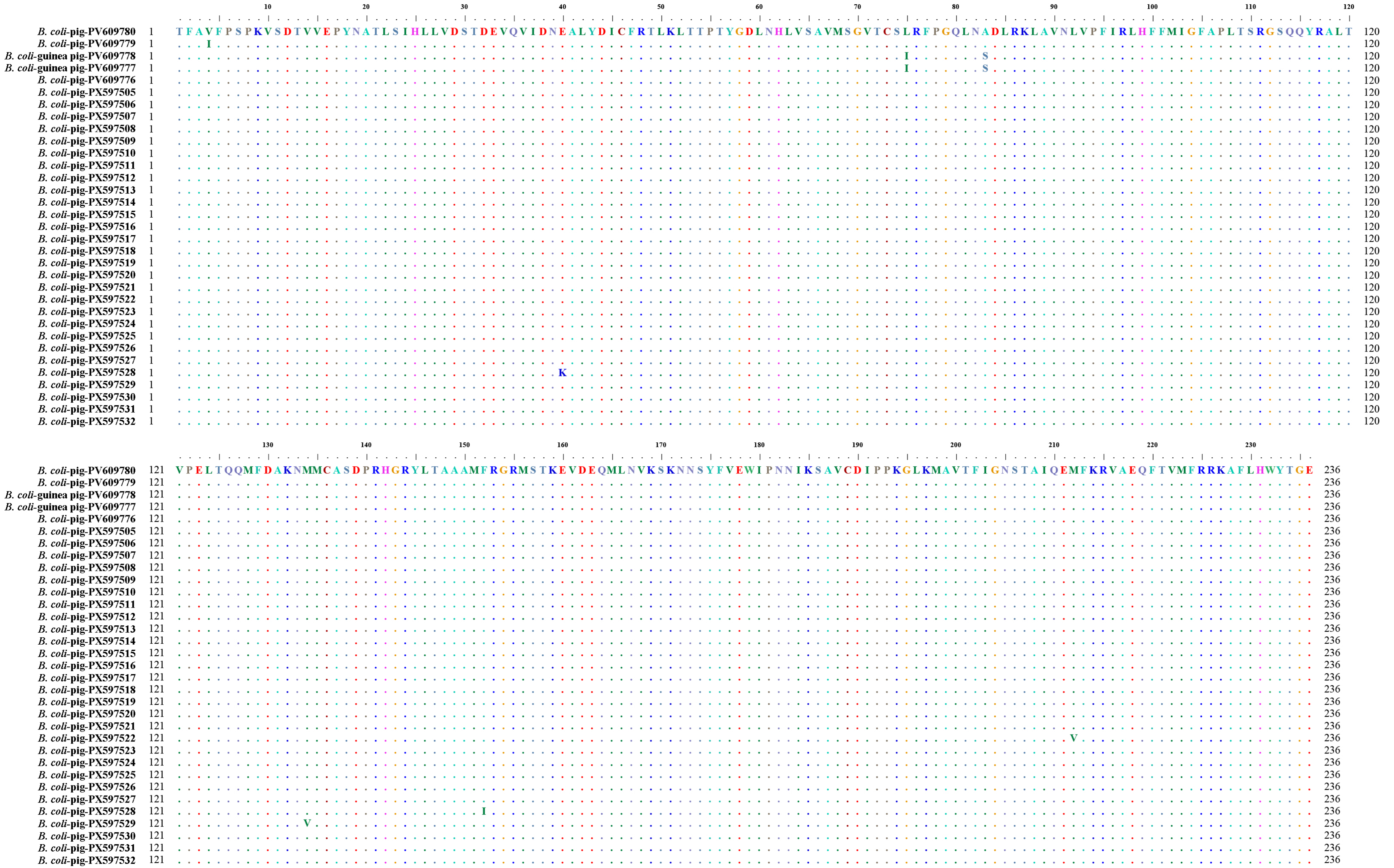

B. coli-pig-PV609780 1 TFAVFPSPKVSDTVVEPYNATLSIHLLVDSTDEVQVIDNEALYDICFRTLKLTTPTYGDLNHLVSAVMSGVTCSLRFPGQLNADLRKLAVNLVPFIRLHFFMIGFAPLTSRGSQQYRALT 120
B. coli-pig-PV609779 1 I 120
B. coli-guinea pig-PV609778 1 I S 120
B. coli-guinea pig-PV609777 1 I S 120
B. coli-pig-PV609776 1 120
B. coli-pig-PX597505 1 120
B. coli-pig-PX597506 1 120
B. coli-pig-PX597507 1 120
B. coli-pig-PX597508 1 120
B. coli-pig-PX597509 1 120
B. coli-pig-PX597510 1 120
B. coli-pig-PX597511 1 120
B. coli-pig-PX597512 1 120
B. coli-pig-PX597513 1 120
B. coli-pig-PX597514 1 120
B. coli-pig-PX597515 1 120
B. coli-pig-PX597516 1 120
B. coli-pig-PX597517 1 120
B. coli-pig-PX597518 1 120
B. coli-pig-PX597519 1 120
B. coli-pig-PX597520 1 120
B. coli-pig-PX597521 1 120
B. coli-pig-PX597522 1 120
B. coli-pig-PX597523 1 120
B. coli-pig-PX597524 1 120
B. coli-pig-PX597525 1 120
B. coli-pig-PX597526 1 120
B. coli-pig-PX597527 1 120
B. coli-pig-PX597528 1 K 120
B. coli-pig-PX597529 1 120
B. coli-pig-PX597530 1 120
B. coli-pig-PX597531 1 120
B. coli-pig-PX597532 1 120
B. coli-pig-PV609780 121 VPELTQQMFDAKNMMCASDPRHGRYLTAAAMFRGRMSTKEVDEQMLNVKSKNNSYFVEWIPNNIKSAVCDIPPKGLKMAVTFIGNSTAIQEMFKRVAEQFTVMFRRKAFLHWYTGE 236
B. coli-pig-PV609779 121 236
B. coli-guinea pig-PV609778 121 236
B. coli-guinea pig-PV609777 121 236
B. coli-pig-PV609776 121 236
B. coli-pig-PX597505 121 236
B. coli-pig-PX597506 121 236
B. coli-pig-PX597507 121 236
B. coli-pig-PX597508 121 236
B. coli-pig-PX597509 121 236
B. coli-pig-PX597510 121 236
B. coli-pig-PX597511 121 236
B. coli-pig-PX597512 121 236
B. coli-pig-PX597513 121 236
B. coli-pig-PX597514 121 236
B. coli-pig-PX597515 121 236
B. coli-pig-PX597516 121 236
B. coli-pig-PX597517 121 236
B. coli-pig-PX597518 121 236
B. coli-pig-PX597519 121 236
B. coli-pig-PX597520 121 236
B. coli-pig-PX597521 121 236
B. coli-pig-PX597522 121 V 236
B. coli-pig-PX597523 121 236
B. coli-pig-PX597524 121 236
B. coli-pig-PX597525 121 236
B. coli-pig-PX597526 121 236
B. coli-pig-PX597527 121 236
B. coli-pig-PX597528 121 I 236
B. coli-pig-PX597529 121 V 236
B. coli-pig-PX597530 121 236
B. coli-pig-PX597531 121 236
B. coli-pig-PX597532 121 236
